# Genomic Analysis of Abnormal DNAM Methylation in Parathyroid Tumors

**DOI:** 10.1155/2022/4995196

**Published:** 2022-07-16

**Authors:** Qing Li, Yonghao Li, Ximei Sun, Xinlei Zhang, Mei Zhang

**Affiliations:** Department of General Surgery, The First Affiliated Hospital of Shandong First Medical University &Shandong Provincial Qianfoshan Hospital, No 16766 Jingshi Road, Jinan, Shandong, China

## Abstract

**Background:**

Parathyroid tumors are common endocrine neoplasias associated with primary hyperparathyroidism. Although numerous studies have studied the subject, the predictive value of gene biomarkers nevertheless remains low.

**Methods:**

In this study, we performed genomic analysis of abnormal DNA methylation in parathyroid tumors. After data preprocessing, differentially methylated genes were extracted from patients with parathyroid tumors by using *t*-tests.

**Results:**

After refinement of the basic differential methylation, 28241 unique CpGs (634 genes) were identified to be methylated. The methylated genes were primarily involved in 7 GO terms, and the top 3 terms were associated with cyst morphogenesis, ion transport, and GTPase signal. Following pathway enrichment analyses, a total of 10 significant pathways were enriched; notably, the top 3 pathways were cholinergic synapses, glutamatergic synapses, and oxytocin signaling pathways. Based on PPIN and ego-net analysis, 67 ego genes were found which could completely separate the diseased group from the normal group. The 10 most prominent genes included POLA1, FAM155 B, AMMECR1, THOC2, CCND1, CLDN11, IDS, TST, RBPJ, and GNA11. SVM analysis confirmed that this grouping approach was precise.

**Conclusions:**

This research provides useful data to further explore novel genes and pathways as therapeutic targets for parathyroid tumors.

## 1. Introduction

Parathyroid cancer is a common endocrine disease characterized by excessive secretion of the parathyroid hormone [1]. The molecular pathogenesis of parathyroid tumors has already been partly elucidated; Heppner and Carling have reported that inactivating somatic mutations of tumor suppressor genes multiple endocrine neoplasia type 1 (MEN1), RET, and HRPT2/CDC73 have been identified in parathyroid tumors [2, 3]. Loss of menin, the protein encoded by the oncosuppressor gene MEN1, is characterized by a genetic background of parathyroid tumors [4]. This is called MEN1 syndrome [4].

It has been demonstrated that epigenetic modifications are not only involved in embryogenesis but also in cell fate reprogramming [5–7]. As epigenetic modifications are present in all human cancers, they cooperate with genetic alterations to drive given cancer phenotypes [8]. Furthermore, the abnormal methylation of cytosine phosphate guanine (CpG) in gene-promoter islands has been investigated in MEN1 syndrome cancer [9]. In benign and malignant parathyroid tumors, despite the clear usefulness in performing large-scale DNA methylation analysis, few studies have been published that describe abnormal global methylation on CpG gene-promoter islands. Global promoter methylation by LINE-1 does not differ from levels detected in normal glands. This differs from most other cancers, which display global DNA hypomethylation [10, 11].

Different CpG sites located near promoter regions of more than 14,000 genes have been screened by Starker and Collaborators, using a model composed of normal, benign, and malignant parathyroid tissues (3 normal parathyroid tissues, 14 adenomas, and 7 carcinomas) [12]. A gradient of CpG hypermethylation levels, ranging from normal tissues to adenomas and carcinomas, was identified in a subset of the genes [12]. These genes were involved in key pathways linked to parathyroid tumors. Specific genes such as RIZ1, APC, RASSF1A, CDKN2A/*p*16 and CDKN2B/*p*15, RB1, WT1, GATA4, PYCARD, SFRP1, SFRP2, and SFRP4 were found to be hypermethylated; this was in line with the hypothesis that cell cycle, transcription, and WNT pathways would represent biological processes that were deranged [12]. They would, in turn, be found in the onset of parathyroid neoplasms [12].

It is important to note that gene markers based solely on expression are still not reliable [13]. However, using cutting-edge computational methods and genomics data, significant markers can be identified. This can be done by integrating gene expression profiles with protein-protein interaction maps. Thus, in this study, we developed a workflow to identify significant methylation of genes that were functionally associated with diseases. We did this so that feature selection could maximize prediction performance [14].

## 2. Methods

### 2.1. Collection of DNA Methylation Data

DNA methylation data for parathyroid tumors (accession no. GSE64412) [15] were accessed from the Gene Expression Omnibus database (GEO, http://www.ncbi.nlm.nih.gov/gds), in the National Center for Biotechnology Information. The microarray data used the GPL11154 platform (Illumina HiSeq 2000 *Homo sapiens*; Illumina Inc., San Diego, CA, USA). GSE64412 included 38 samples: 13 sporadic (non-MEN1) parathyroid adenomas, 12 MEN1-parathyroid tumors, 4 parathyroid carcinomas, and 9 normal parathyroid tissues.

### 2.2. Data Preprocessing and Identification of Differentially Methylated Genes

A methylation-identification algorithm found in Genelibs (http://www.genelibs.com) was utilized. Raw microarray data containing 1954997 CpGs sites were filtered down in order to better focus our study. Sites were eliminated when they met the following criteria: (i) distance from CpG to single-nucleotide polymorphism (SNP) was less than or equal to 2; (ii) minor allele frequency (MAF) was less than 0.05; (iii) cross-hybridized probes were found, or on sex chromosomes. 1925786 CpGs were kept for further study. The DNA methylation microarray data were then processed using the Lumi package (bioconductor.org/packages/release/bioc/html/lumi) [16, 17]. Data were normalized via the *β*-mixture quantile normalization method [18].

In this study, *β* values have been represented graphically, both the diseased group and the normal group. The percentage of methylation used the following formula: methylated/(methylated + unmethylated); results range between 0 and 1, where zero represented fully unmethylated genes and one represented fully methylated. Subsequently, the absolute value of the difference in mean *β* values was calculated, termed A. *T*-tests were then employed to identify the differentially methylated CpGs and identified at the threshold of *P* < 0.05 and *A*>0.01.

In order to decrease the number of nonvariable sites, further filtering steps were performed. All sites with methylation scores were >50; the differential expression *p* value was <0.001, and the absolute value of >3 remained. Only CpGs termed A with a value of ≥17 (and corrected *p* value <0.05) were applied. This was done in order to detect substantial methylation differences.

### 2.3. Hierarchical Clustering Analysis

Hierarchical clustering is an analytical tool applied to discover the closest associations that exist between gene profiles and specimens [19]. Generally, cancers have similar methylation profiles that tend to cluster together. To analyze whether these differentially methylated CpGs would segregate into two distinct clusters, unsupervised hierarchical clustering was conducted using Euclidian distance and average linkage criteria [20]. The matrix of mean *β*-value levels was then formed between the parathyroidoma and normal samples.

### 2.4. Gene Ontology (GO) Analysis of Differentially Methylated Genes

Gene ontology (GO) was applied to analyze the main function of differentially expressed genes (DEGs), the key functional classification used by the National Center of Biotechnology Information (NCBI). In the present study, Fisher's exact test was used to ascertain the GO category; furthermore, *P* values were corrected using the false discovery rate (FDR) along with the Benjamini & Hochberg method [21]. Functional terms with an FDR <0.01 and gene count >10 were considered to be statistically significant.

### 2.5. Pathway Enrichment Analysis of Differentially Methylated Genes

Pathway analysis was used to find significant pathways of the DEGs according to the Kyoto Encyclopedia of Genes and Genomes (KEGG) [22]. In the present study, Fisher's exact test was used to extract significant pathways, and the threshold of significance was defined by FDR. Significant pathways were selected according to the thresholds of FDR <0.01 and gene count >10.

### 2.6. Identification of the Ego Genes in the Methylation Correlation Network

The Search Tool for the Retrieval of Interacting Genes (STRING) database (http://www.bork.embl-heidelberg.de/STRING) is a global resource used for analyzing gene-gene interaction [23]. In the present study, STRING was utilized to identify the correlations between the differentially methylated genes.

First, a network of all human gene interactions was obtained from the STRING database; a special subnetwork that contained differentially methylated genes and parathyroid adenoma genes (CDC73, MEN1, CCND1, RET) was then used for further analysis. The absolute value of the Pearson coefficient was calculated: interaction pairs with a *p* value of 0.05 defined the difference expression network [24]. Finally, the weight value of interaction in the network was calculated using the following formula (EgoNet algorithm) [25]:

(1) $$\begin{array}{c} \omega_{ij}=\frac{\sqrt{\log\;p_{i}+\log\;p_{j}}}{\sqrt{2^{*}\max_{i\in V}\left|\log\;p_{i}\right|}}\left(\text{ifcor}\left(i,j\right)\geq \delta \right)\omega_{ij},=0\left(ifcor\left(i,j\right)<\delta \right), \end{array}$$

where V represents the node set in the network.

The topological analysis of the differential expression network was measured by four indicators: degree, closeness, betweenness, and transitivity (a measure of the clustering coefficient). In order to identify the methylation markers, the context likelihood of relatedness (CLR) was applied to the algorithm to square weight and then to form an adjacency matrix. Genes were sorted in descending order based on the importance of genes in the netweb (g (i) = z-score).

### 2.7. Support Vector Machine Analysis

The support vector machine (SVM) is a machine learning method commonly used in pattern recognition for binary classification [26]. In this study, SVM was used to test whether ego genes could completely separate the diseased group from the normal group and to further verify the feasibility of our analysis methods.

The normal group and the diseased group were together termed the total population. In accordance with the ratio of 6 : 4, the total population was randomly divided into an experimental group and a testing group. SVM C-classification was then performed using linear kernel and 5-fold cross-validation. The experimental group served as the basis for classification, and the testing group as the basis for regression [27].

## 3. Results

### 3.1. Identification of Differentially Methylated Genes

**Figure 1:**
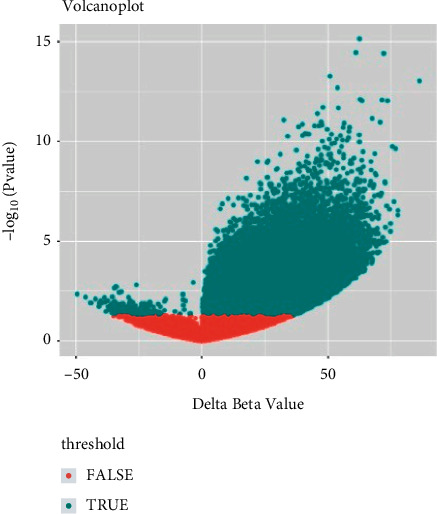
Volcano plot exhibiting the methylation data of 29 parathyroid tumors and 9 normal samples. X-axis represents the mean methylation difference between parathyroid tumor and normal samples. Y-axis represents the log-transformed *P* values. A total of 192613 CpG sites were considered to be significantly differentially methylated, indicated in blue.

Following quality control and normalization to (i) remove probes with an SNP-CpG distance of ≤2, (ii) remove probes with a minimum allelic frequency of <0.05, and (iii) demonstrate cross-hybridization, a total of 1925786 methylated CpGs remained. A volcano plot exhibiting the distribution of the 1925786 methylated CpGs is presented in Figure 1. Among these 1925786 methylated CpGs, 192613 of them (representing 26926 genes) were differentially methylated, where the absolute value of the mean *β*-value was >0.05, and the *P* value <0.05. A total of 192340 of the CpGs were hypermethylated, and 273 CpGs were hypomethylated in the disease group.

Subsequently, these 192613 methylated CpGs were subjected to further filtering. 31207 unique CpGs (covering 13798 genes) met the following conditions: [1] a methylation score >50; [2] differential expression *P* value < 0.001, and [3] absolute value > 3. The only CpGs with the absolute value of the mean *β*-value difference were ≥17, with the corrected *P* value <0.05.28241. CpGs (covering 634 genes) were selected for further analysis. According to various studies, CDC73, MEN1, CCND1, and RET were shown to be parathyroid adenoma susceptible genes [28–31]. Their *P* values are demonstrated in Table 1.

### 3.2. Hierarchical Clustering Analysis

**Figure 2:**
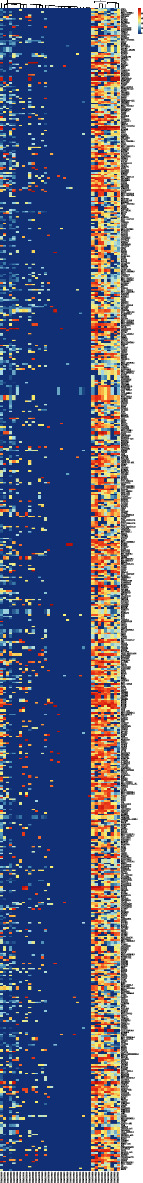
Hierarchical clustering analysis of significantly differentially methylated CpGs between parathyroidoma and normal samples. DNA methylation across the 28241 sites in each of the samples was analyzed by this method. Each row is an individual CpG site and each column is a different sample. The color gradation from green to red denotes low to high DNA methylation, with *β*-values ranging from 0 (no methylation; green) to 1 (complete methylation; red).

To further explore the changes in the methylation levels, cluster analysis was conducted. The cluster heat map is demonstrated in Figure 2. In this figure, it is observed that there were distinctive methylation patterns in the parathyroidoma and normal samples, further segregating the samples into two distinct groups.

### 3.3. GO Enrichment

**Figure 3:**
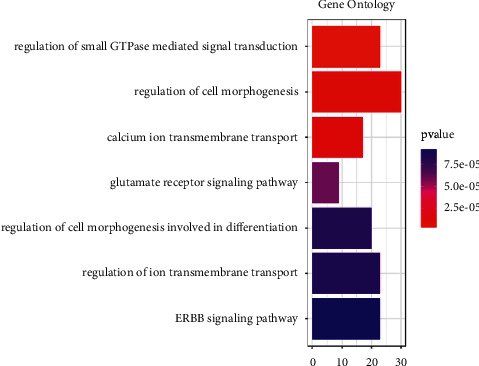
Differentially methylated genes identified between parathyroidoma and normal samples were functionally classified via gene ontology analysis. The most enriched terms, which satisfied the criteria of FDR <0.01 and gene count >10, are represented.

To determine the primary functions of the differentially methylated genes, a comprehensive gene ontology (GO) analysis was performed. As shown in Figure 3, 7 signaling pathways were significantly enriched by the differentially methylated genes, including regulation of small GTPase mediated signal transduction, regulation of cell morphogenesis, calcium ion transmembrane transport, glutamate receptor signaling pathway, regulation of cell morphogenesis involved in differentiation, regulation of ion transmembrane transport, and ERBB signaling pathway. These GO terms were sorted in ascending order based on FDR value. Overall, the GO terms were mainly involved in cell morphogenesis, ion transport, and GTPase signal.

### 3.4. KEGG Pathway Analysis

Pathway enrichment analysis of the differentially methylated genes was conducted given the KEGG pathway database. This analysis yielded 48 pathways.  Table 2 lists the top 10 pathways. These pathways were sorted in ascending order based on FDR value. The top 3 significantly enriched pathways were cholinergic synapses, glutamatergic synapses, and the oxytocin signaling pathway. The oxytocin signaling pathway was associated with CCND1.

### 3.5. Ego Analysis

**Figure 4:**
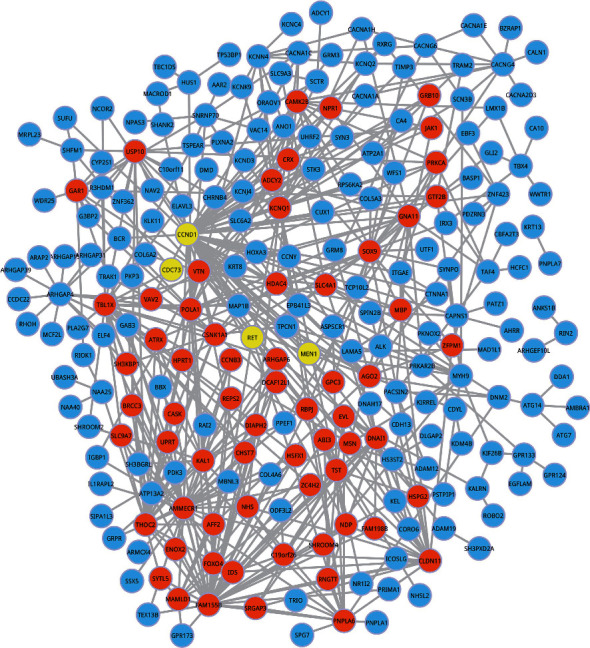
Protein-protein interaction network constructed from differentially methylated genes. Nodes colored in orange are gene-associated ego genes. Yellow nodes represent parathyroid adenoma susceptibility genes. It is paramount to explicate that CCDN 1 and RET are parathyroid adenoma susceptibility genes as well ego genes.

Given the goal of analyzing the association between the differentially methylated genes, STRING software was used to establish the PPI network (PPIN). A network of all human gene interactions containing 787896 interaction pairs (16730 genes) was obtained from STRING. A total of the 793 interaction pairs (the target-PPI) were extracted from the PPIN. The target-PPI included differentially methylated genes as well as parathyroid adenoma susceptible genes (CDC73, MEN1, CCND1, RET). This covered 299 genes. The weight value of the interaction in the network was calculated, and genes were sorted in descending order based on topological degree. The top 30% were selected as ego genes. A total of 67 ego genes were obtained (Figure 4). The differential expression of the 67 genes ensures complete separation between the normal and parathyroid tumor populations. Genes with higher z-scores were given greater importance. The top 10 of the 67 ego genes are shown in Table 3.

### 3.6. Support Vector Machine Analysis

The use of machine learning in medical diagnosis is gaining currency. This is mainly because the effectiveness of classification and recognition systems has significantly improved, helping medical experts in diagnosing diseases [32, 33]. In this paper, C-classification was performed using linear kernel and 5-fold cross-validation. Assessment indicators were AUC (the area under receiver operating characteristic curve), accuracy (classification accuracy), MCC (Mathews' correlation coefficient), specificity (TNR, true-negative rates), and sensitivity (TPR, true-positive rates). The results of the analysis are as follows: AUC = 0.9123, accuracy = 99.29%, MCC = 0.8549, specificity = 83%, and sensitivity = 92%. Thus, the methods used in this study could completely separate the diseased group from the normal one.

## 4. Discussion

Analysis of DNA methylation data has been widely used to identify abnormally methylated genes associated with tumors. It has also enabled the extraction of targets for therapeutic strategies. In this study, the pathogenesis of parathyroid tumors was analyzed by means of bioinformatics. This included the detection of differentially methylated genes, gene ontology (GO), pathway enrichment, PPI construction, and ego genes identification. Potential mechanisms of parathyroid tumors have been thus revealed, providing novel insights into parathyroid diagnosis and therapy.

In general, the genetic background of parathyroid tumors is characterized by the loss of two oncosuppressor genes. First, a loss of menin, the protein encoded by the MEN1 gene, takes place. This occurs in the parathyroid lesions found in multiple endocrine neoplasia type 1 (MEN1), [34]. Second is a loss of parafibromin, encoded by the CDC73/HRPT2 gene. This occurs in hyperparathyroidism-jaw tumors (HPT-JT) [35].

The heterogeneity of parathyroid tumors' biological and clinical presentation may be due to epigenetic deregulation [36, 37]. As mentioned, epigenetic modifications at DNA and chromatin levels are present in all human cancers [38–40]. Epigenetic regulation is achieved through multiple regulatory pathways unifying sequence-specific, DNA-binding transcription factors, ATP-dependent, nucleosome remodeling, and long, noncoding RNA and DNA methylation [41–44].

Our model aimed at finding the diseased genes by means of the following underlying assumption: if the majority of neighbors of a central disease gene are also disease genes, then its other neighbors are likely to be involved in the disease pathway. This model's benefit is that it can find hidden genes that would otherwise show no significance by themselves but are nevertheless clustered in a subnetwork module. It is in this subnetwork where collectively the genes are highly predictive of disease status. Furthermore, machine-learning techniques can be used to assess the association between ego-networks and clinical outcomes.

Based on pathway analyses, pathways of cholinergic synapses, glutamatergic synapses, and oxytocin signaling were considered the most important. The oxytocin signaling pathway in particular was associated with CCND1. On the other hand, cholinergic and glutamatergic synapses were closely related to the regulation of nerve excitement and inhibition. In GO analysis, the differentially methylated genes were enriched in 7 GO terms, which were mainly involved in cell morphogenesis, ion transport, and GTPase signal. Recent studies suggest that there is a cross-regulation between morphogenesis and EMT processes. Aberrant activation of these coregulators can drive different stages of cancer progression, including tumor invasion, cell spread, and metastatic colonization and growth [45] The ion (such as Ca^2+^, K^+^, Fe^3+^, and Cl^−^) and channel proteins play important roles in tumor progression. These membrane ion transport systems have great potential as diagnostic biomarkers and therapeutic targets in tumor treatment [46–48]. GTPase-related signaling pathways are widely abnormally activated in tumors and are involved in the regulation of tumor cell growth, metastasis, and drug resistance [49]. However, there are few studies on the relationship between these three cell functions and the tumorigenesis of parathyroid tumors, suggesting that further research in this area should be conducted in the future.

Following the implementation of the PPIN and ego-net, 67 ego genes were found, which completely separates normal from parathyroid tumor populations. The top 10 of the 67 ego genes were *POLA1*, *FAM155 B, AMMECR1*, *THOC2, CCND1*, *CLDN11*, *IDS*, *TST*, *RBPJ*, and *GNA11*. At present, there have been studies conducted on *CCND1*, but little research on the other genes. It is reported that *AMMECR1* is associated with growth, bone, and heart alterations; *CLDN11* is an epigenetic biomarker for malignancy, and *THOC2* mutations are related to X-linked intellectual disability [50–52]. *POLA1* is the catalytic subunit of DNA polymerase and plays an essential role in the initiation of DNA replication [53]. *FAM155 B* is predicted to be a multipass membrane protein with unknown function. *IDS* is involved in the lysosomal degradation of heparan sulfate and dermatan sulfate [54]. *TST* is mainly localized to mitochondria and catalyzes the conversion of thiosulfate and cyanide to thiocyanate and sulfite [55]. As molecular switches in regulating the notch response, transcription factor *RBPJ* involves in the regulation of the progression of a variety of tumors, such as colorectal cancer [56], lung cancer [57], hepatocellular carcinoma [58], glioblastoma [59, 60], and prostate cancer [61]. As a member of the G protein family, *GNA11* plays an important role in tumor progression [62]. Research shows that CCND1 and POLA1 are potential targets of miR-206 and may share a common regulatory network [63]. The interrelationship between these genes has not been studied.

Global promoter methylation by CCND1 often displays differentially expressed DNA hypomethylation, especially when compared to normal tissue [64]. Different CpG sites located near promoter regions of more than 14,000 predicted genes were screened. Both CCND1 and CCND2 have been previously reported as deregulated in many cancers [65, 66]. Furthermore, they represent an alternative marker to study epigenetic modifications [66]. The reversibility of these changes makes them prime targets for therapeutic manipulations. A number of small molecules targeting chromatin-based mechanisms are currently being tested in clinical trials [67]. However, the results of this study were based on a limited clinical sample, which limited the accuracy of the data. The role of the above biomarkers still needed to be verified with more samples. In addition, we did not analyze the difference of MEN1 in adenomas, carcinomas, or sporadic tumors due to the limitation of sample size. Importantly, our conclusions still require experimental confirmation, not just bioinformatics data analysis. The expression of MEN1 in subgroups of parathyroid tumors is still worthy of further analysis.

In conclusion, we have evaluated the performance of EgoNet in human protein-protein interaction networks. This method has not only successfully identified CCND1 from significant ego-networks but has also detected several novel targets, such as POLA1, FAM155 B, AMMECR1, and THOC2. We expect that EgoNet can be widely used to infer novel biomarkers for phenotypic prediction of many human diseases.

## Figures and Tables

**Table 1:** *P* values of susceptible parathyroid adenoma genes.

ID	*p* value	Symbol
1173651	0.003161659	RET
1173667	0.003246324	RET
1173680	7.16E−05	RET
1173684	0.000342777	RET
1173722	0.002885583	RET
1173736	0.002602326	RET
1173749	0.001025146	RET
1173756	0.000703971	RET
1173773	0.000353432	RET
1301532	0.001475949	MEN1
1312429	0.004835513	CCND1
1312477	0.003523037	CCND1
150982	0.001799107	CDC73

**Table 2:** The top 10 key pathways according to KEGG analysis.

Pathway name	*P* value
4725 Cholinergic synapse [PATH:hsa04725]	3.27E−07
4724 Glutamatergic synapse [PATH:hsa04724]	4.63E−07
4921 Oxytocin signaling pathway [PATH:hsa04921]	5.67E−06
4261 Adrenergic signaling in cardiomyocytes [PATH:hsa04261]	4.82E−05
4911 Insulin secretion [PATH:hsa04911]	0.000101911
4713 Circadian entrainment [PATH:hsa04713]	0.0002795
4020 Calcium signaling pathway [PATH:hsa04020]	0.000363009
4912 GnRH signaling pathway [PATH:hsa04912]	0.000829401
4730 Long-term depression [PATH:hsa04730]	0.00111826
5205 Proteoglycans in cancer [PATH:hsa05205]	0.001260031

**Table 3:** The top 10 key ego genes based on ego-net analysis.

Node	z-Score
POLA1	219.1896777
FAM155B	202.6303015
AMMECR1	161.7614615
THOC2	135.7028647
CCND1	132.8239023
CLDN11	128.873499
IDS	126.8873715
TST	125.1721484
RBPJ	117.4724126
GNA11	115.4442218

## Data Availability

The data supporting the conclusions of this paper are included within the manuscript.

## Abbreviations

MEN1: Multiple endocrine neoplasia type 1
CpG: Cytosine phosphate guanine
GEO: Gene expression omnibus
SNP: Single-nucleotide polymorphism
MAF: Minor allele frequency
GO: Gene ontology
DEGs: Differentially expressed genes
NCBI: National Center of Biotechnology Information
FDR: False discovery rate
KEGG: Kyoto Encyclopedia of Genes and Genomes
STRING: Search Tool for the Retrieval of Interacting Genes
CLR: Context likelihood of relatedness
SVM: Support vector machine
GO: Gene ontology
PPIN: PPI network
AUC: The area under receiver operating characteristic curve
Accuracy: Classification accuracy
MCC: Mathews' correlation coefficient
TNR: True-negative rates
TPR: True-positive rates
HPT-JT: Hyperparathyroidism-jaw tumors.
